# Microvascular function and oxidative stress in adult individuals with early onset of cardiovascular disease

**DOI:** 10.1038/s41598-020-60766-0

**Published:** 2020-03-17

**Authors:** Hala Shokr, Irundika H. K Dias, Doina Gherghel

**Affiliations:** 1 Vascular Research Laboratory, School of Life and Health Sciences, Aston University, Birmingham, UK; 2 Aston Medical Research Institute, Aston Medical School, Aston University, Birmingham, UK

**Keywords:** Cardiology, Risk factors, Cardiovascular diseases

## Abstract

The current study aims to investigate retinal vascular function and its relationship with systemic anti-oxidative defence capacity in normal individuals versus those with early hypertensive changes according to the current ESC/ESH guidelines. Retinal microvascular function was assessed in 201 participants by means of dynamic retinal vessel analysis. Blood pressure, lipid panel, oxidized (GSH) & reduced glutathione (GSSG) were also evaluated for each participant. Individuals classed as grade 1 hypertension demonstrated higher retinal arterial baseline diameter fluctuation (p = 0.0012), maximum dilation percentage (p = 0.0007), time to maximum constriction (p = 0.0003) and lower arterial constriction slope (p = 0.0131). Individuals classed as high normal and grade 1 hypertension also demonstrated higher time to maximum dilation than individuals classed as optimal or normal. GSH levels correlated negatively with SBP, DBP and MBP values in all participants (p = 0.0010; p = 0.0350 and p = 0.0050) as well as with MBP values in high normal and grade 1 hypertension (p = 0.0290). The levels of GSSG correlated positively with SBP, DBP and MBP values in all participants (p = 0.0410; p = 0.0330 and, p = 0.0220). Our results point to the fact that microvascular alterations can be identifiable at BP values still considered within normal values and go in parallel with the changes observed in the level of oxidative stress.

## Introduction

The assessment of the microvascular function represents an important part in establishing the pathophysiology but also the risk stratification of cardiovascular disease (CVD)^1^. Indeed, endothelial dysfunction, one of the main culprits for the development of atherosclerosis, occurs much earlier at the microvascular than at the macrovascular level^2,3^. Dynamic retinal vessel analysis (DVA) was identified as a useful measure of early changes that signal endothelial dysfunction at the microvascular level. This method can also be used to identify risk for future cardiovascular pathologies in individuals at risk for^4–9^ or already suffering from CVD^10,11^. This is generally possible due to the fact that the retinal microvascular response to flicker provocation is, in part, dependent on nitric oxide (NO) release^12^, and compromised NO homoeostasis is known to be a key factor in endothelial dysfunction at all vascular levels.

The assessment of retinal microvessels is used in research but also in clinical practice for diagnosis and follow-up of various CVD, including hypertension^13,14^. Nevertheless, the overwhelming majority of protocols that look at retinal microvessels in the course of various CVD, use static imaging to detect abnormalities associated with various degrees of pathology. This is, however, useful only when CVD already established itself and not at earlier, pre-clinical stages. In order to determine the risk at earlier stages to allow preventive measures to be adopted, using the assessment of the retinal microvascular function represents a better alternative to structural imaging because it provides integrated and dynamic data to help establishing possible CVD risk.

In certain conditions that accelerate degradation of NO, such as high oxidative stress, microvascular dilation can be severely impaired^15^. In order to counteract such effects, the human body uses various anti-oxidative mechanisms including glutathione. Therefore, any condition associated with low levels of circulating glutathione result in a higher rate of oxidative reactions that contribute towards low NO bioavailability and, consequently, to an impaired microvascular function^5,8,16,17^. We have already shown that retinal microvascular dilation and constriction responses to stress levels are influenced by systemic antioxidant capacity, and circulating markers for CVD risk in healthy individuals with low to moderate cardiovascular risk^7^. Moreover, we have suggested that, by providing an integrated and dynamic analysis of vascular function that is, indeed, specific for each individual, retinal vessel reactivity could also be used for profiling a so-called *individualized vascular risk* for CVD^7,8^. Nevertheless, we have not tested this hypothesis in individuals with early stages of CVD. Therefore, the present study aims to study the retinal vascular function and its relationship with systemic anti-oxidative markers in individuals with various levels of early BP abnormalities as defined according to the 2018 European Society of Cardiology/European Society of Hypertension Guidelines^18^.

## Methods

### Study participants

Healthy individuals over the age of 30 were recruited through advertisements at the Vascular Research Laboratory, Aston University (Birmingham, UK), for inclusion in this prospective study. Ethical approval was sought from the relevant local ethics committee, and written informed consent was received from all participants prior to study enrolment. All experimental protocols were approved by the Aston University’s Ethics Committee and the study was designed and conducted in accordance with the tenets of the Declaration of Helsinki.

Study exclusion criteria were defined as the positive diagnosis of CVD, (coronary artery disease, heart failure, arrhythmia, stroke, transient ischaemic attacks), cerebrovascular disease, peripheral vascular disease, severe dyslipidaemia (defined as plasma triglycerides > 6.00 mmol/l or cholesterol levels > 7.00 mmol/l), diabetes, as well as other metabolic disorders or chronic diseases that required treatment. Individuals treated for systemic hypertension as well as those using other vasoactive medications such as dietary supplements containing vitamins or antioxidants and bronchodilators were also excluded from the study. Potential participants were also screened for ocular diseases and were excluded from the study if they had a refractive error of more than ± 3DS and more than ± 1DC equivalent, intra‐ocular pressure (IOP) greater than 21 mmHg, cataract or any other media opacities, as well as history of intra‐ocular surgery or any form of retinal or neuro‐ophthalmic disease affecting the ocular vascular system. Individuals with sings of hypertensive retinopathy at the initial fundus examination were also excluded.

All the above investigations are standard for our lab^7,8,19–21^.

### General investigations

Participants who met the inclusion criteria were requested to complete a general health history questionnaire, also detailing daily diet, physical activity and alcohol consumption. All study‐related measurements were performed between 8 and 11 am following a 12‐hr overnight fast, which included refraining from alcohol and caffeine.

Standard anthropometric measures of height and weight were recorded to determine body mass index (BMI = weight/height²).

### Blood pressure assessment and patients grouping

Measurements of BP and heart activity were first perdormed in-clinic. They were then confirmed using a computer‐operated ambulatory BP and electrocardiogram (ECG) monitor (Cardiotens‐01, Meditech Ltd, Budapest, Hungary). This device measures BP automatically using an oscillometric method and can store 1000 BP measurements. All subjects maintained their normal activity and were carefully instructed to complete a diary each time their activities changed, or when any chronic medication was taken. The 24‐h BP were later downloaded and analysed using the ‘Medibase’ software program (Meditech). The average systolic (SBP) as well as diastolic BP (DBP) measurements were calculated for the 24‐h interval and for both the active and passive periods of the recording. The average readings for SBP and DBP were then used to calculate the mean BP (MBP) using the formula: MBP = 2/3 × DBP + 1/3 × SBP. At least 80% of the programmed recordings were required for a diurnal curve to be considered in the present analysis^22^.

Using the 24-h SBP and DBP values, study participants were then stratified into four subcategories, ‘’optimal ‘’ “normal”, “high normal” and ‘’Grade I” as recommended by the 2018 European Society of Cardiology/European Society of Hypertension arterial hypertension Guidelines^18^.

### Dynamic retinal vessel analysis

**Figure 1:**
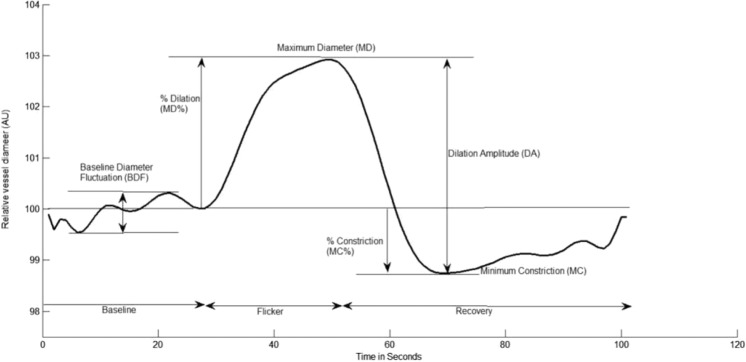
Graphical presentation of the dynamic vessel response profile displaying the parameters calculated and used in analysis. (DA) calculated as (MD-MC). (MD%) calculated as the percent increase from baseline to MD. (MC%) calculated as the percent constriction below baseline following MD^42^.

Retinal vessel reactivity was assessed using the dynamic retinal vessel analyser (DVA, IMEDOS GmbH, Jena, Germany) in accordance with an established protocol^23^. All measurements were performed in a temperature-controlled environment (22 °C) following pupil dilation with 1% Tropicamide (Chauvin Pharmaceuticals Ltd, UK) and were taken from the inferior temporal vessel branches approximately one and a half disc diameters from the optic nerve head of one unselected eye. The flicker stimulation protocol involves a 350‐second continuous diameter recording along short 1 mm sections of the retinal vasculature, the duration of which included a 50‐second baseline measurement followed by three successive cycles of flicker stimulation distinguished as a 20‐second stimulus (opto‐electronically generated at 12.5 Hz) and an 80‐second recovery period (still illumination)^7^. The following vessel reactivity and time-course parameters were determined for each flicker cycle and then averaged over the 3 cycles, with the artery and vein regarded separately as follows: the average baseline diameter and range of maximum and minimum baseline vessel diameters (baseline diameter fluctuation, BDF); the maximum vessel dilation diameter during flicker stimulation expressed as a percentage change relative to baseline diameter (MD%) and the time taken in seconds to reach the maximum diameter (tMD); the maximum vessel constriction diameter during the postflicker recovery period expressed as a percentage change relative to baseline diameter (MC%) and the time taken in seconds to reach the maximum vessel constriction diameter (tMC); the overall dilation amplitude (DA) calculated as the difference between MD and MC; and the baseline-corrected flicker response (BCFR) used to describe the overall dilation amplitude after normalizing for fluctuations in baseline diameters (DA-BDF). In addition, the arterial (A) and venous (V) dilation slopes (Slope_AD/VD_ = (MD − baseline diameter)/tMD) and constriction slopes (Slope_AC/VC_ = (MC − MD)/tMC) were also calculated (Fig. 1)^6^.

**Figure 2:**
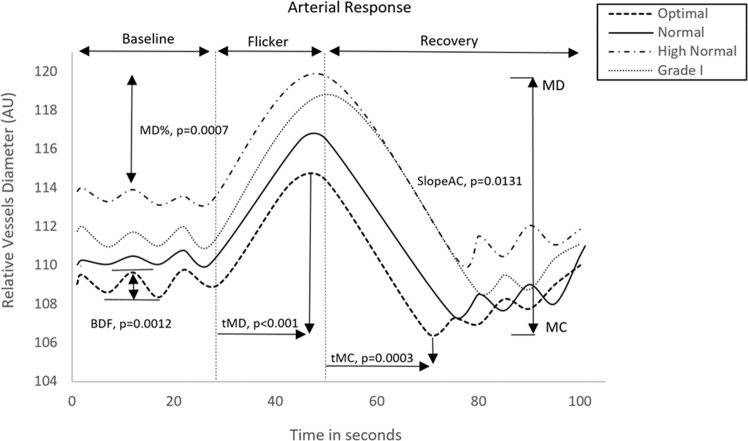
Comparison of retinal arterial response profile across groups. AU, arbitrary units; BDF, baseline diameter fluctuation calculated as the maximum range in vessel diameter during first 30 seconds of baseline readings; MD%, calculated as the percentage change in vessel diameter from baseline to maximum following onset of flicker; tMD, time to reach maximum diameter during flicker; tMC, time to reach maximum constriction post flicker; Slope_AC,_,calculated as (MC-MD)/(tMC).

### Biomarker assays

Bloods samples drawn from the ante‐cubital fossa vein on the morning of the appointment were collected into standard EDTA Vacutainer^®^ tubes and assessed immediately for fasting triglycerides (TG), plasma total cholesterol (CHOL) and high‐density lipoprotein cholesterol (HDL‐C) using the Reflotron Desktop Analyser (Roche Diagnostics, UK). Low‐density lipoprotein cholesterol (LDL‐C) values were calculated as per the Friedewald equation^24^.

### Measurement of GSH and oxidized glutathione (GSSG)

Initial processing of blood GSH and GSSG levels were assessed by the GSH recycling assay as detailed previously^21^. Briefly, a 30 *μ*l aliquot of EDTA blood was pretreated with 33.3 *μ*l of 100 mg/ml 5‐sulfosalicylic acid (SSA), 936.7 *μ*l sodium phosphate buffer (pH 7.5) to release GSH via cellular disruption and protein precipitation. The sample was centrifuged at 13, 000 rpm for 5 min, and the supernatant was stored at −80 °C for further analyses. Based on previous reports of sample stability, assays were conducted within 2 months of collection^25^. The GSH levels [t‐GSH – (2 × GSSG)] and the redox index (defined as the GSH/GSSG ratio) were determined according to an established enzymatic recycling assay^26,27^ that involves the oxidation of GSH by the sulphydryl reagent 5,5′‐dithio‐bis(2‐nitrobenzoic acid) (DTNB) to form the yellow derivative 5′‐thio‐2‐nitrobenzoic acid (TNB), which can be measured spectrophotometrically at 412 nm.

Any GSSG formed was recycled to GSH by glutathione reductase (GSR) in the presence of nicotinamide adenine dinucleotide phosphate‐oxidase (NADPH). For the measurement of GSSG levels, samples were pretreated with, 2‐vinylpyridine (2VP) to derivatize GSH without interfering with GSR reaction. For the determination of analyte concentrations in the samples, GSH standards were prepared from 0 to 80 *μ*m in increments of 20 *μ*m and GSSG standards were prepared from 0 to 10 *μ*m in increments of 1 *μ*m with the same final concentrations of SSA (1%) as in the samples. Standard curves were generated using a linear regression program (Microsoft Excel, Microsoft Corporation, USA). As the microplates were read at 0, 1, 2, 3 and 5 min, the change in optical density or absorbance at different times points expressed against GSH or GSSG concentration (net reaction rate = slope * GSH or GSSG concentration + intercept) was used to determine GSH or GSSG concentrations in the samples (net rate − intercept/slope * dilution factor). The blood GSH and GSSG concentrations measured in this study were in good agreement with literature data in control patients in the ranges of 150–1500 *μ*m and 1–500 *μ*m, respectively^5,8,23,28,29^. This suggests that the experimental conditions reported in this study are suitable for the analysis of total glutathione and glutathione disulphide concentrations in whole blood. The validity and reliability of the spectrophotometric method of detection has also previously been established^30^.

### Sample size and statistical analysis

Based on previous studies, a change of 30% with a SD of 2.5% in retinal vessels reactivity was shown to be significant^8,31^. As the study design was multifactorial in nature, it was calculated that a sample size of *n* = 42 in each group was sufficient to provide 95% power at an alpha level of 0.05.

All analyses were performed using Statistica® software (version 13.3, StatSoft Inc., Tusla, OK, USA). Distributions of continuous variables were determined by the Shapiro-Wilks test. In cases where normality of the data could not be confirmed appropriate data transformations were made or non-parametric statistical alternatives were used. Univariate associations were determined using Pearson’s (normally distributed data) or Spearman’s method (non-normally distributed data), and forward stepwise regression analyses were performed to test the influence of clinical parameters and circulating markers on the measured vascular reactivity variables. Differences between groups were subsequently assessed using one-way ANOVA or ANCOVA, as appropriate, followed by Tukey’s post hoc analysis. *P*-values of <0.05 were considered significant^5–7^.

## Results

A total number of 218 participants were initially screened for study inclusion of which 17 individuals were excluded based on the quality of retinal vascular image analysis. The remaining 201 participants (93 men and 108 women) were included in the final analysis and classified in one of 4 study groups using the current ESC/ESH Guidelines: group 1 (optimal BP: 56 individuals 22 men and 34women), group 2 (normal BP: 54 individuals, 35 men and 19 women), group 3 (high normal BP: 44 individuals, 28 men and 16 women) and group 4 (grade 1 hypertension: 47 individuals, 28 men and 19 women).

Table 1 shows the clinical characteristics of the study population. Although the first group were significantly younger than group 4, there were no statistically significant differences between the study groups with regards to gender, BMI and circulating levels of glucose, t-Chol and HDL-Chol between the study groups (all p > 0.05). Nevertheless, as expected, there were statistically significant differences between the 4 study groups with regards to SBP, DBP and MBP (all p < 0.0001). In addition, individuals classed as high normal and grade 1 hypertension demonstrated higher levels of circulating TG (p = 0.0021, ANCOVA) and GSSG (p = 0.0111, ANCOVA) and lower levels of GSH (p = 0.0060, ANCOVA). Moreover, individuals classed as grade 1 hypertension demonstrated higher levels of LDL-C (p = 00265, ANCOVA) than the rest of the study groups.With regards to arterial and venous retinal vascular reactivity parameters as characterized in Tables 2 and 3, all values reported are based on averaged data across three flicker cycles with the artery and vein regarded separately.With regards to the measured retinal arteriolar parameters, after controlling for all influential covariates identified by multivariate regression analysis, and independent of age, individuals classed as grade 1 hypertension demonstrated significantly higher BDF (p = 0.0012, Fig. 2), MD% (p = 0.0007, Fig. 2), tMC (p = 0.0003, Fig. 2) and lower Slope_AC_ (p = 0.0131, Fig. 2) than the rest of the study groups. In addition, individuals classed as high normal and grade 1 hypertension also demonstrated higher tMD than individuals classed as optimal or normal (p < 0.0001, Fig. 2).There were no significant differences between participants with regard to measured venous parameters (all p > 0.05, Table 3).

**Table 1:** Summary of the systemic characteristics of the study participants.

Variable	Optimal (1) (Mean + SD)	Normal (2) (Mean + SD)	High-Normal (3) (Mean + SD)	Grade 1 (4) (Mean + SD)	*p-*value	Post –Hoc
Number	56	54	44	47	—	—
Age (years)	40.25(1.80)	42.67(1.87)	46.50(2.06)	47.60(2.03)	0.0260*	—
SBP (mmHg)	109.11(0.79)	124.59(0.82)	131.95(0.90)	145.31(0.90)	0.0000*	1 < 2 < 3 < 4
DBP (mmHg)	67.38(1.07)	74.71(1.11)	81.17(1.22)	87.59(1.22)	0.0000*	1 < 2 < 3 < 4
HR (bpm)	66.69(1.27)	67.61(1.32)	68.48(1.46)	70.24(1.46)	0.3149	—
IOP (mmHg)	14.31(0.82)	15.15(0.85)	16.76(0.93)	16.30(0.93)	0.1870	—
MAP (mmHg)	81.29(1.30)	91.33(1.35)	98.09(1.48)	104.35(1.47)	0.0000*	1 < 2 < 3 < 4
BMI (kg/m^2^)	25.32(0.90)	26.88(1.027)	27.89(0.99)	28.21(0.88)	0.0808	—
Glucose	4.77(0.12)	4.80(0.12)	4.90(0.14)	5.01(0.13)	0.4875	—
TG (mmol/L)	1.01(0.06)	1.15(0.06)	1.29(0.07)	1.43(0.07)	0.0021*	1 = 2 > 3,4 3 = 4
CHOL	4.53(0.13)	4.38(0.13)	4.62(0.15)	4.80(0.14)	0.1875	—
HDL-C (mmol/L)	1.21(0.07)	1.13(0.07)	1.19(0.08)	1.16(0.07)	0.6838	—
LDL-C (mmol/L)	2.92(0.15)	3.06(0.16)	3.27(0.18)	3.59(0.17)	0.0265*	1 = 2 = 3 < 4
GSH(umol)	712.37(68.79)	601.47(74.18)	412.52(77.17)	428.63(83.43)	0.0060*	1 = 2 > 3,4 3 = 4
GSSG(umol)	36.15(7.92)	55.53(8.54)	62.49(9.75)	76.67(9.47)	0.0111*	1 = 2 < 3,4 3 = 4

Abbreviations: SBP: systolic blood pressure; DBP: diastolic blood pressure; HR: heart rate; IOP: intraocular pressure; BMI: body mass index; TG: triglycerides; CHOL: total cholesterol; HDL-C: high-density lipoprotein cholesterol; LDL-C: low-density lipoprotein; GSSG: oxidized glutathione; GSH: reduced glutathione. *Significant p-values are indicated where p < 0.05 was considered significant.

**Table 2:** Summary of retinal arterial vascular function parameters.

Variable	Optimal BP (1) (Mean + SD)	Normal BP (2) (Mean + SD)	High-Normal BP (3) (Mean + SD)	Grade 1 BP (4) (Mean + SD)	p-value Anova/Ancova	Post-Hoc
Baseline	109.00(2.23)	110.42(2.48)	113.81(2.97)	111.77(4.45)	0.08319	—
BDF	5.36(0.40)	5.75 (0.42)	6.62(0.46)	8.12(0.46)	0.0012*	1 = 2 = 3 < 4
BCFR	4.62(0.40)	3.85(0.48)	4.08(0.53)	5.74(0.81)	0.2646	—
MD	114.22(2.44)	115.54(2.72)	119.86(3.25)	118.81(4.87)	0.1194	—
tMD	12.76(0.698)	14.77(0.735)	18.04(0.842)	23.10(0.79)	0.0000*	1 = 2 < 3 < 4
MD %	5.46(0.53)	4.31(0.58)	5.17(0.57)	7.31(0.513)	0.0007*	1 = 2 = 3 < 4
MC	106.14(2.39)	107.39(2.39)	110.35(3.18)	108.60(4.76)	0.2053	
tMC	23.85(1.07)	25.40(1.109)	26.77(1.22)	30.84(1.22)	0.0003*	1 = 2 = 3 < 4
MC%	−4.31(0.282)	−3.63(0.293)	−4.042(0.32)	−3.23(0.32)	0.1696	—
DA	9.11(0.66)	9.04(0.735)	9.46(0.88)	11.32(1.35)	0.4951	—
Slope_AD_	0.69(0.066)	0.57(0.069)	0.37(0.08)	0.51(0.075)	0.1539	—
Slope_AC_	−0.45(0.07)	−0.44(0.07)	−0.48(0.08)	−0.74(0.08)	0.0131*	1 = 2 = 3 > 4

Abbreviations: ANOVA, analysis of variance; ANCOVA, analysis of covariance; Baseline, baseline diameter; BDF, baseline diameter fluctuation; BCFR, Baseline corrected flicker response; tMD, time to reach MD; MD (%), percent dilation; tMC, time to reach MC; MC (%), percent constriction; DA, dilation amplitude (difference between MD and MC during flicker) Slope_AD_, slope of arterial dilation; Slope_AC_, slope of arterial constriction. *Significant *p-*values are indicated where *p* < 0.05 was considered significant.

**Table 3:** Summary of retinal venous vascular function parameters.

Variable	Optimal BP (1) (Mean + SD)	Normal BP (2) (Mean + SD)	High-Normal BP (3) (Mean + SD)	Grade 1 BP (4) (Mean + SD)	p-value Anova/Ancova
BL	146.41(3.93)	144.11(4.08)	151.22(4.50)	143.30(4.44)	0.5542
BDF	6.08(0.50)	4.769(0.60)	5.21(0.75)	6.51(0.93)	0.1913
BCFR	4.42(0.41)	4.21(0.43)	4.86(0.47)	3.88(0.47)	0.4718
MD	153.77(4.07)	152.77(4.18)	161.22(4.63)	152.10(4.59)	0.4623
tMD	19.97(0.79)	20.56(0.94)	21.18(1.198)	22.05(1.46)	0.0525
MD%	5.28(0.35)	5.44(0.37)	5.42(0.40)	6.10(0.41)	0.2231
MC	143.04(3.89)	141.74(4.038)	148.13(4.45)	140.56(4.40)	0.6051
tMC	34.30(1.29)	31.10(1.43)	32.90(1.70)	32.50(2.53)	0.4114
MC%	−1.76(0.20)	−1.57(0.22)	−1.48(0.31)	−1.90(0.38)	0.7218
DA	9.79(0.72)	8.61(0.73)	11.22(0.84)	11.16(0.83)	0.1069
Slope_VD_	0.39(0.03)	0.38(0.04)	0.36(0.05)	0.41(0.056)	0.8740
Slope_VC_	−0.48(0.10)	−0.39(0.11)	−0.498(0.12)	−0.83(0.12)	0.0663

Abbreviations ANOVA, analysis of variance; ANCOVA, analysis of covariance; Baseline, baseline diameter; BDF, baseline diameter fluctuation; BCFR, Baseline corrected flicker response; tMD, time to reach MD; MD (%), percent dilation; tMC, time to reach MC; MC (%), percent constriction; DA, dilation amplitude (difference between MD and MC during flicker) Slope_VD_, slope of venous dilation; Slope_VC_, slope of venous constriction. *Significant p-values are indicated where p < 0.05 was considered significant.

### Correlations between vascular and systemic circulatory parameters

Univariate analysis revealed that the whole blood GSH levels correlated significantly and negatively with SBP, DBP and MBP values in all participants (r = −0.25, p = 0.0010; r = −0.17, p = 0.0350 and r = −0.22, p = 0.0050, as well as with MBP values in those classed as high normal and with grade 1 hypertension (r = −0.38, p = 0.0290) but not in those classed as having optimal or normal BP (p > 0.05). In addition, the levels of whole blood GSSG correlated significantly and positively with SBP, DBP and MBP values in all participants (r = 0.16, p = 0.0410; r = 0.17, p = 0.0330 and r = 0.18, p = 0.0220,but not in any groups separately (all p > 0.05).

There were no correlations between the levels of GSH and retinal vascular parameters in any of the study groups (all p > 0.05). However, GSSG levels correlated significantly and positively with artery BDF (r = 030, p = 0.0370) and negatively with Slope_AC_ only in individuals classed as having an optimal BP level (r = −0.37, p = 0.0200).

## Discussions

In the present study, we have examined the differences in retinal vascular function between individuals with normal and early stages of abnormal BP according to the current ESC/ESH guidelines. We have also looked at the relationship between systemic BP, retinal vessel reactivity and systemic antioxidant defense capacity in each of the study groups. Our results showed, for the first time, that individuals graded as having high normal BP values or grade 1 hypertension displayed abnormal dilatory and constrictor responses to flicker light stimulation in retinal arteries but not in the retinal veins when compared to those classed as having optimal or normal BP values.

Hypertension is a polygenic disease; however, endothelial dysfunction and enhanced oxidative stress are among the established modifiable risk factors for this disease^32^. Alteration of vascular reactivity characterized by augmented contractility and impaired relaxation is a prominent feature in hypertension. Moreover, in essential hypertension, NO availability is reduced earlier, resulting in early vascular endothelial dysfunction^33^. Indeed, our relatively young patients with high normal BP and grade 1 hypertension according to the current ESC/EHS guidelines demonstrated abnormal retinal microvascular dilation and constriction after stimulation with flickering light.

It has already been demonstrated that pre-hypertension is associated with retinal microvascular alterations early in life^34^. Nevertheless, it seems that these alterations can also be identifiable at BP values previously considered within normal values. Indeed, our study participants classed as having high normal BP values and without additional risks for CVD, have also exhibited signs of microvascular dysfunction at the retinal level. At these BP values, treatments for hypertension are not yet indicated; however, in the light of the above observations, additional measures to prevent further damage may be considered. Moreover, ESC/ESH also recommends treating at lower BP levels in very high-risk patients with a high–normal BP and established CVD^18^. Nevertheless, the potential impact of pushing for an earlier diagnosis is multiple, including pressures on the health system and economy^35^. Therefore, a careful consideration should be given to the actual benefits of treatment in each individual separately, rather than in a given risk category. At present, all the new biomarkers for CVD risk drive the shift towards personalized medicine. Therefore, due to its personalised approach, retinal vessel reactivity can be used for profiling individualized vascular risk by providing an integrated and dynamic analysis of vascular function specific for each individual, therefore, aiding a personalised management of abnormal BP values.

As already mentioned above, increased oxidative stress is an important risk factor for the development of hypertension. Among other defense mechanisms, glutathione represents a major redox buffer^36^. It has been already shown that GSH significantly lower and the GSSG is higher in the red blood cells in patients with hypertension but not in normal individuals^37^. Similarly, we have also shown that whole-blood GSH level were lower and GSSG level were higher in individuals classed as high normal and grade 1 hypertension. These results show for the first time that such changes may actually occur at a much earlier stage than previously noticed and go in parallel in the changes observed in the retinal microvasculature, confirming that, in the presence of abnormal BP levels, the pathological inhibition of vascular relaxation involves not only NO production by endothelial cells but also a glutathione-dependent bioavailability of NO^38^. In addition, we also demonstrated that GSSG correlated with retinal arterial baseline and constriction characterisation parameters only in individuals with optimal BP levels and without additional CVD risks. It is very interesting that, although the levels of antioxidative products were abnormal in individuals with higher BP levels, the link between redox status and retinal vascular reactivity was lost in these groups. This observation links, however, to our previous results in in individuals with low to moderate CVD risk, indicating that in addition to other processes, antioxidant mechanisms support normal NO levels during flicker provocation at the retinal microvascular level^7^. However, in the present study we could not identify such relationship in individuals with higher levels of BP. As the effects of radical oxygen species (ROS) on vascular tone is dependent upon the concentration and type of species, as well as on the type of vascular beds and various experimental conditions^39^, it is possible that these effects were not visible in our specific setup. Indeed, it has been proposed that there is a lack of sensitive methods to accurately evaluate the oxidative stress in the human cardiovascular system, especially when it comes to establishing the role redox stress in the small artery vasculopathy of human hypertension^40^.

## Conclusions

Abnormalities of microvascular dysfunction may be an important risk factor for systemic hypertension. In addition, excessive oxidative stress is associated results in cardiovascular diseases by impairing endothelial function and, consequently, microvascular dysfunction. This complex interlink cannot be ignored and measures to address both dysfunctions should be applied. This is especially important since, despite significant advancement in understanding the pathophysiology of human hypertension and the large number of antihypertensive drugs, strict BP control is still proving insufficient for the prevention of future vascular complications, the so-called “Hypertension Paradox”^41^. Therefore, more research is necessary to develop specific mechanism-based personalised therapies that will address the vascular redox pathobiology^40^. These therapies will possibly need to be applied at much earlier stage when abnormalities are only functional and can still be reversed.

## Data Availability

The data that support the findings of this study are available on request from the corresponding author.

## References

[1] Rizzoni D (2011). How to assess microvascular structure in humans. High. Blood Press. Cardiovascular Prevention..

[2] Gariano RF, Gardner TW (2005). Retinal angiogenesis in development and disease. Nature..

[3] Brown MD (2009). Endothelial ageing: molecular mechanisms and functional significance. Exp. Physiol..

[4] Heitmar R, Blann AD, Cubbidge RP, Lip GY, Gherghel D (2010). Continuous retinal vessel diameter measurements: the future in retinal vessel assessment?. Invest. Ophth Vis. Sci..

[5] Patel SR (2011). Abnormal retinal vascular function and lipid levels in a sample of healthy UK South Asians. Brit J. Ophthalmol..

[6] Seshadri S, Ekart A, Gherghel D (2016). Ageing effect on flicker‐induced diameter changes in retinal microvessels of healthy individuals. Acta Ophthalmol..

[7] Seshadri S (2015). Systemic circulatory influences on retinal microvascular function in middle‐age individuals with low to moderate cardiovascular risk. Acta Ophthalmol..

[8] Patel SR (2012). Abnormal retinal vascular reactivity in individuals with impaired glucose tolerance: a preliminary study. Invest. Ophth Vis. Sci..

[9] Nagel E, Vilser W, Fink A, Riemer T, Lanzl I (2006). Blood pressure effects on retinal vessel diameter and flicker response: a 1.5-year follow-up. Eur. J. Ophthalmol..

[10] Pemp B (2009). Correlation of flicker-induced and flow-mediated vasodilatation in patients with endothelial dysfunction and healthy volunteers. Diabetes care..

[11] Lanzl IM (2011). Dynamic retinal vessel response to flicker in age‐related macular degeneration patients before and after vascular endothelial growth factor inhibitor injection. Acta ophthalmol..

[12] Dorner GT (2003). The ocular hemodynamic response to nitric oxide synthase inhibition is unaltered in patients with early type I diabetes. Graefes Arch. Clin. Exp. Ophthalmol..

[13] McGeechan K (2009). Meta-analysis: retinal vessel caliber and risk for coronary heart disease. Ann. Intern. Med..

[14] Wong TY (2001). Retinal microvascular abnormalities and their relationship with hypertension, cardiovascular disease, and mortality. Surv. Ophthalmol..

[15] Cines DB (1998). Endothelial cells in physiology and in the pathophysiology of vascular disorders. Blood,J. Am. Soc. Hematology..

[16] Frisard MI (2007). Aging, resting metabolic rate, and oxidative damage: results from the Louisiana Healthy Aging Study. J. Gerontol. A Biol. Sci. Med. Sci..

[17] Salmon AB, Richardson A, Pérez VI (2010). Update on the oxidative stress theory of aging: does oxidative stress play a role in aging or healthy aging?. Free. Radic. Bio Med..

[18] Williams B (2018). 2018 ESC/ESH Guidelines for the management of arterial hypertension: The Task Force for the management of arterial hypertension of the European Society of Cardiology (ESC) and the European Society of Hypertension (ESH). Eur. Heart J..

[19] Kotliar KE (2011). Dynamic retinal vessel response to flicker in obesity: A methodological approach. Microvasc. Res..

[20] Garhofer G (2010). Use of the retinal vessel analyzer in ocular blood flow research. Acta Ophthalmol..

[21] Gherghel D, Mroczkowska S, Qin L (2013). Reduction in blood glutathione levels occurs similarly in patients with primary-open angle or normal tension glaucoma. Invest. Ophth Vis. Sci..

[22] Gherghel D, Hosking SL, Armstrong R, Cunliffe IA (2007). Autonomic dysfunction in unselected and untreated primary open angle glaucoma patients: a pilot study. Ophthalmic Physiol. Opt..

[23] Nagel E, Vilser W, Lanzl I (2004). Age, blood pressure, and vessel diameter as factors influencing the arterial retinal flicker response. Invest. Ophth Vis. Sci..

[24] Friedewald WT, Levy RI, Fredrickson DS (1972). Estimation of the concentration of low-density lipoprotein cholesterol in plasma, without use of the preparative ultracentrifuge. Clin. Chem..

[25] Jones DP (1998). Glutathione measurement in human plasma: evaluation of sample collection, storage and derivatization conditions for analysis of dansyl derivatives by HPLC. Clinica Chim. acta..

[26] Tietze F (1969). Enzymic method for quantitative determination of nanogram amounts of total and oxidized glutathione: applications to mammalian blood and other tissues. Anal. Biochem..

[27] Smith MA (1998). Cytochemical demonstration of oxidative damage in Alzheimer disease by immunochemical enhancement of the carbonyl reaction with 2, 4-dinitrophenylhydrazine. J. Histochem. Cytochem..

[28] Rossi R (2002). Blood glutathione disulfide: *in vivo* factor or *in vitro* artifact?. Clin. Chem..

[29] Gherghel D, Griffiths HR, Hilton EJ, Cunliffe IA, Hosking SL (2005). Systemic reduction in glutathione levels occurs in patients with primary open-angle glaucoma. Invest. Ophth Vis. Sci..

[30] Rahman I, Kode A, Biswas SK (2006). Assay for quantitative determination of glutathione and glutathione disulfide levels using enzymatic recycling method. Nat. Protoc..

[31] Garhöfer G (2004). Reduced response of retinal vessel diameters to flicker stimulation in patients with diabetes. Brit J. Ophthalmol..

[32] Abdilla N (2007). Impact of the components of metabolic syndrome on oxidative stress and enzymatic antioxidant activity in essential hypertension. J. Hum. Hypertens..

[33] Bruno RM, Masi S, Taddei M, Taddei S, Virdis A (2018). Essential hypertension and functional microvascular ageing. High. Blood Press. Cardiovascular Prevention..

[34] Imhof K, Zahner L, Schmidt-Trucksäss A, Hanssen H (2016). Association of body composition and blood pressure categories with retinal vessel diameters in primary school children. Hypertens. Res..

[35] Goel, H., Tayel, H., & Nadar, S. K. Aiming higher in hopes to achieve lower: the European Society of Cardiology/European Society of Hypertension versus the American College of Cardiology/American Heart Association guidelines for diagnosis and management of hypertension. *Nature*. 635–638 (2019). 10.1038/s41371-019-0227-6 31431680

[36] Robaczewska J (2016). Role of glutathione metabolism and glutathione-related antioxidant defense systems in hypertension. J. Physiol. Pharmacol..

[37] Muda P (2003). Homocysteine and red blood cell glutathione as indices for middle-aged untreated essential hypertension patients. J. Hypertens..

[38] Rybka J (2011). Glutathione-related antioxidant defense system in elderly patients treated for hypertension. Cardiovasc. Toxicol..

[39] Lee MY, Griendling KK (2008). Redox signaling, vascular function, and hypertension. Antioxid. Redox Signal..

[40] Touyz RM, Montezano AC, Rios F, Widlansky ME, Liang M (2017). Redox stress defines the small artery vasculopathy of hypertension: How do we bridge the bench-to-bedside gap?. Circ. Res..

[41] Chobanian AV (2009). The hypertension paradox—more uncontrolled disease despite improved therapy. N. Engl. J. Med..

[42] Mroczkowska S (2012). Coexistence of macro‐and micro‐vascular abnormalities in newly diagnosed normal tension glaucoma patients. Acta Ophthalmol..

